# PSMA-PET-CT zum primären Staging von Patienten mit fortgeschrittenem Prostatakarzinom

**DOI:** 10.1007/s00066-020-01732-7

**Published:** 2021-01-15

**Authors:** Cedric Curt Cappel, Denise Dopcke, Jürgen Dunst

**Affiliations:** Klinik für Strahlentherapie Kiel, UKSH, Arnold-Heller-Str. 3, Hs. L, 24105 Kiel, Deutschland

## Hintergrund

Im Jahr 2016 erhielten 58.780 Menschen in Deutschland die Diagnose eines Prostatakarzinoms, davon befanden sich fast 40 % bereits in den fortgeschrittenen Stadien III und IV nach UICC. Während im Stadium III ohne Vorliegen von regionalen oder Fernmetastasen die tumorspezifische 5‑Jahres-Überlebensrate noch bei etwa 100 % liegt, beträgt sie im Stadium IV nur noch 50 % [2]. Als Grundlage für die Therapieplanung werden daher möglichst exakte Staging-Untersuchungen benötigt. Die aktuellen Leitlinien empfehlen bei einem lokal fortgeschrittenen Prostatakarzinom eine CT- bzw. MRT-Untersuchung der Beckenorgane sowie eine Skelettszintigraphie mittels ^99m^Tc-basierten Tracern [3]. Eine funktionelle Bildgebung mittels Positronenemissionstomographie (PET) oder PET-CT-Hybridverfahren ist derzeit nur zur Rezidivdiagnostik nach Anstieg des PSA-Werts trotz kurativer Primärtherapie indiziert. Mit der ^68^Ga-PSMA-PET-CT steht jedoch seit einigen Jahren ein Verfahren zur funktionellen Darstellung von Prostatagewebe zur Verfügung. Als Zielstruktur dient dabei das prostataspezifische Membranantigen PSMA, welches in hohem Maße von Prostatazellen und insbesondere Prostatakarzinomen exprimiert wird [4]. PSMA ist darüber hinaus auch in anderen Drüsengeweben, wie den Speicheldrüsen, sowie in Duodenum, Kolon und Nieren vorhanden, allerdings in weit geringerem Maße [5]. Für die Visualisierung mittels PET dient der synthetische Inhibitor PSMA-11 als Ligand, der einen Komplex mit dem Positronenstrahler^68^Ga bildet [6]. Präklinisch zeigten sich dabei eine gute Kontrastierung von Tumorgewebe [6] sowie eine geringe intraindividuelle Variabilität der Traceraufnahme [7]. In klinischen Studien konnte die PSMA-PET-CT zwar mit einer hohen Sensitivität und Spezifität überzeugen [8], jedoch stammen die bisherigen Ergebnisse größtenteils aus retrospektiven Fallstudien [9]. Um den diagnostischen Nutzen der ^68^Ga-PSMA-PET-CT im primären Staging des Prostatakarzinoms verlässlich und im Vergleich zum bisherigen diagnostischen Standard beurteilen zu können, wurde schließlich die proPSMA-Studie als randomisierte, kontrollierte klinische Studie gestartet [1].

## Patientenkollektiv und Methoden

Für diese zweiarmige Studie, durchgeführt an zehn Zentren in Australien, wurden zwischen dem 22. März 2017 und dem 2. November 2018 insgesamt 300 Männer rekrutiert und randomisiert einem der beiden Studienarme zugeteilt. Eingeschlossen wurden hierbei lediglich Patienten, welche ein histopathologisch gesichertes Prostatakarzinom und zusätzlich einen PSA Wert ≥20 ng/ml, einen ISUP-Grad ≥3 bzw. ein cT3-Stadium oder höher aufwiesen. Zudem mussten sie für eine radikale Prostatektomie oder eine Radiotherapie mit kurativer Intention infrage kommen. Die Probanden wurden zufällig im Verhältnis 1:1 auf die beiden Studienarme verteilt: In der Kontrollgruppe erhielten diese ein „first-line imaging“, bestehend aus einer CT von Becken und Abdomen, sowie eine ^99m^Tc-SPECT-CT. In der experimentellen Gruppe bestand das „first-line imaging“ aus einer ^68^Ga-PSMA-11-PET-CT unter Nutzung eines standardisierten Protokolls. Im Folgenden wurde bei den Probanden, welche im „first-line imaging“ drei oder mehr uneindeutige Herde mit dem Verdacht auf Fernmetastasen aufwiesen, ein „cross-over imaging“ mittels der jeweils anderen bildgebenden Methode durchgeführt, um mögliche Nachteile für den Patienten auszugleichen. Im Follow-up nach 6 Monaten wurde die Bildgebung unter Berücksichtigung der ursprünglichen, randomisiert zugeteilten Gruppe wiederholt und wenn nötig erneut um ein „cross-over imaging“ erweitert.

Als primärer Endpunkt dieser Studie wurde die Genauigkeit des im „first-line imaging“ angewendeten Verfahrens in Bezug auf die Identifizierung pelviner Lymphknotenmetastasen oder Fernmetastasen festgelegt. Diese Genauigkeit wurde mithilfe der „area under the curve“ (AUC) der ROC-Kurve eingeschätzt. Die AUC spiegelt dabei ein Maß für Sensitivität und Spezifität wider. Die Hypothese wurde definiert als eine AUC der PSMA-PET-CT, welche mindestens 10 % größer als die der konventionellen Bildgebungsgruppe sein sollte. Als Referenzstandard, inwiefern ein Befund als positiv hinsichtlich einer pelvinen Lymphknotenmetastase oder Fernmetastase zu werten ist, wurden verschieden vordefinierte Kriterien herangezogen und nach 6 Monaten beurteilt. Harte Kriterien waren die histopathologische Sicherung eines Adenokarzinoms der Prostata und die Veränderung einer Knochenläsion zu einer sklerotischen oder blastischen im „follow-up imaging“. Zusätzlich gab es weitere weiche Kriterien.

## Ergebnisse

Es konnte herausgestellt werden, dass in der Gruppe der PSMA-PET-CT eine um 27 % absolut höhere AUC und damit höhere Genauigkeit vorlag als in der Gruppe der konventionellen Bildgebung. Sensitivität wie auch Spezifität waren somit deutlich höher unter Anwendung der PSMA-PET-CT. Auch in der Analyse der Subgruppen bestätigte sich dies und zeigte eine um absolut 32 % höhere Genauigkeit bei der Erkennung von pelvinen Lymphknotenmetastasen sowie eine 22 % absolut höhere Genauigkeit bei Fernmetastasen im Vergleich zur konventionellen Bildgebung. Zusätzlich wurden durch die konventionelle Bildgebung mehr uneindeutige Befunde generiert. Dies war in der Kontrollgruppe bei 23 % und in der PSMA-PET-CT-Gruppe nur bei 7 % der Fall. Außerdem wurde der Einfluss der Bildgebungsart auf mögliche Therapieänderungen untersucht. Bei 28 % mit „first-line imaging“ mittels PSMA-PET-CT wurde ein mittlerer oder großer Effekt auf den Therapieverlauf im Gegensatz zu 15 % der Patienten in der Kontrollgruppe festgestellt. Auch die Strahlenbelastung der Probanden wurde in dieser Studie betrachtet. Es zeigte sich, dass diese durch das „first-line imaging“ mit konventioneller Bildgebung, bestehend aus Becken/Abdomen-CT und Szintigraphie, um insgesamt 10,9 mSv höher und somit mehr als doppelt so hoch lag wie mit der PSMA-PET-CT.

## Schlussfolgerung der Autoren

Die Genauigkeit der PSMA-PET-CT übertrifft deutlich die einer CT-Untersuchung bzw. einer Szintigraphie und ermöglicht dabei weniger uneindeutige Befunde. Der Einsatz einer PSMA-PET-CT führt in mehr Fällen zur Änderungen der Behandlungsstrategie.

Auch die Kosten seien im Vergleich zu CT und Szintigraphie günstiger. Die überlegene Genauigkeit der PSMA-PET-CT führe zu insgesamt weniger bildgebenden Untersuchungen.

Unter allen betrachteten Gesichtspunkten stellt die PSMA-PET-CT einen geeigneten Ersatz für die konventionelle CT mit Szintigraphie beim Staging von Patienten mit High-risk-Prostatakarzinom und kurativer Therapieintention dar.

## Kommentar

Obwohl PET-CT-basierte Staginguntersuchungen bei vielen Tumorentitäten vielversprechende Studienergebnisse bieten, ist ihre Anwendung im klinischen Alltag noch auf sehr spezielle Indikationen begrenzt. Im Falle des Prostatakarzinoms, der zweithäufigsten Krebserkrankung des Mannes in Deutschland [2], hat die PSMA-PET-CT einzig im Rahmen der Rezidivdiagnostik einen Platz in den aktuellen Leitlinien gefunden. Bisher fehlten aber im Rahmen des primären Stagings bei Patienten mit einem Hochrisikoprofil Daten aus randomisierten, kontrollierten Studien [9]. Daher leistet die proPSMA-Studie einen wertvollen Beitrag für die Einschätzung der PSMA-PET-CT zur Verbesserung des diagnostischen Prozederes. Hofman et al. konnten die diagnostische Überlegenheit der PSMA-PET-CT im Sinne einer deutlich höheren Sensitivität für regionale Lymphknoten- und Fernmetastasen bei mindestens gleichwertiger Spezifität darstellen. Weiterhin lieferte die PSMA-PET-CT mehr eindeutige Befunde als das konventionelle, CT-basierte Verfahren und führte bei ca. 28 % der Patienten zur Änderung der geplanten Therapie. Die PSMA-PET-CT hat somit eine unmittelbare therapeutische Konsequenz, nicht nur im Sinne einer Änderung der Therapieintention. Sie ermöglicht im Falle oligometastasierter Erkrankungen auch den Einsatz von bildgesteuerten Hochpräzisionsbestrahlungen („image-guided radiotherapy“ [IGRT]), welche bereits positive Ergebnisse in klinischen Studien geliefert haben [10].

Die proPSMA-Studie konnte allerdings, bedingt durch das Studiendesign, keinen Effekt auf das Überleben oder die Lebensqualität der Patienten ableiten. Die häufigere Änderung des Therapieregimes mag hierfür ein Indikator sein, jedoch lässt sich dies durch die erhobenen Daten keineswegs belegen. Aufgrund der ohnehin vergleichsweise hohen 5‑ und 10-Jahres-Überlebensraten bei Prostatakarzinomen sind dringend weitere klinische Studien nötig, um die Langzeiteffekte der verbesserten Diagnostik durch die PSMA-PET-CT abschätzen und eine Aufnahme in die Leitlinien erreichen zu können.

Die hohe Inzidenz des Prostatakarzinoms in Deutschland stellt zudem ein weiteres Problem für den routinemäßigen Einsatz der PSMA-PET-CT dar: Bei einer Inzidenz von jährlich etwa 145 Fällen pro 100.000 männlichen Einwohnern fallen statistisch gesehen bundesweit etwa 22.500 Patienten in die Hochrisikogruppen III und IV nach UICC, was die Kapazitäten der PET-Diagnostik bei Weitem übersteigt, zumal die PSMA-PET-CT derzeit nur an spezialisierten Zentren angeboten wird. Hofman et al. spekulieren zwar, dass die Gesamtkosten durch die verbesserte Sensitivität und dadurch geringere Zahl an erforderlichen Kontrolluntersuchungen insgesamt eher sinken würden. Jedoch steht dies den enormen Anschaffungskosten für ein flächendeckendes Angebot an PSMA-PET-CT-Untersuchungen gegenüber. Realistischerweise werden derzeit PSMA-PET-CT-Untersuchungen nur in Einzelfällen von den gesetzlichen Krankenkassen bezahlt.

Ein weiterer Schwachpunkt der Studie ist der Vergleich des empfohlenen Verfahrens mit einem CT-basierten Staging, dem die Kontrollgruppe unterzogen wurde. Die deutschen Leitlinien schließen nämlich für das Staging explizit auch MRT-Untersuchungen der Beckenorgane ein, die möglicherweise durch den verbesserten Weichteilkontrast auch eine höhere Sensitivität erreichen könnten. Der entsprechende Vergleich wurde von den Autoren aber nicht adressiert und sollte unbedingt mit weiteren Studien untersucht werden. PET-MRT-Hybridverfahren sind zwar in manchen Zentren bereits etabliert und wurden auch mit dem ^68^Ga-PSMA-11-Tracer in Studien erprobt [11], jedoch liegt ihre Verfügbarkeit noch deutlich unter der einer PET-CT. Auch die von Hofman et al. propagierte Verminderung der Strahlendosis durch eine PET-CT-Untersuchung sollte mit Vorsicht aufgenommen werden, weil ein MRT-basiertes Staging mit Sicherheit die einfachere Methode ist zur Reduktion der Strahlendosis.

## Fazit

Die proPSMA-Studie führte bei Patienten mit Prostatakarzinomen einen sorgfältig geplanten und dann auch realisierten Vergleich der PSMA-PET-CT-Technologie mit den herkömmlichen CT-basierten Stagingverfahren durch. Obwohl noch weitere Studien zur Auswirkung auf Überlebensraten und Lebensqualität nötig sind, belegt sie zweifelsfrei die diagnostische Überlegenheit des PET-Verfahrens. Sie leistet damit einen wichtigen Beitrag zu deren langfristiger Etablierung bei der Diagnostik des fortgeschrittenen Prostatakarzinoms und damit zur Optimierung der Patientenversorgung.

*Cedric Curt Cappel, Denise Dopcke *

*und Jürgen Dunst, Kiel*
